# HEAL: High-Frequency Enhanced and Attention-Guided Learning Network for Sparse-View CT Reconstruction

**DOI:** 10.3390/bioengineering11070646

**Published:** 2024-06-25

**Authors:** Guang Li, Zhenhao Deng, Yongshuai Ge, Shouhua Luo

**Affiliations:** 1 Jiangsu Key Laboratory for Biomaterials and Devices, School of Biological Science and Medical Engineering, Southeast University, Nanjing 210096, China; liguang@seu.edu.cn (G.L.); dzh_dengzhenhao@163.com (Z.D.); 2 Research Center for Medical Artificial Intelligence, Shenzhen Institute of Advanced Technology, Chinese Academy of Sciences, Shenzhen 518055, China; 3 Paul C Lauterbur Research Center for Biomedical Imaging, Shenzhen Institute of Advanced Technology, Chinese Academy of Sciences, Shenzhen 518055, China; 4 Key Laboratory of Biomedical Imaging Science and System, Chinese Academy of Sciences, Shenzhen 518055, China

**Keywords:** CT imaging, sparse-view, dual-domain network

## Abstract

X-ray computed tomography (CT) imaging technology has become an indispensable diagnostic tool in clinical examination. However, it poses a risk of ionizing radiation, making the reduction of radiation dose one of the current research hotspots in CT imaging. Sparse-view imaging, as one of the main methods for reducing radiation dose, has made significant progress in recent years. In particular, sparse-view reconstruction methods based on deep learning have shown promising results. Nevertheless, efficiently recovering image details under ultra-sparse conditions remains a challenge. To address this challenge, this paper proposes a high-frequency enhanced and attention-guided learning Network (HEAL). HEAL includes three optimization strategies to achieve detail enhancement: Firstly, we introduce a dual-domain progressive enhancement module, which leverages fidelity constraints within each domain and consistency constraints across domains to effectively narrow the solution space. Secondly, we incorporate both channel and spatial attention mechanisms to improve the network’s feature-scaling process. Finally, we propose a high-frequency component enhancement regularization term that integrates residual learning with direction-weighted total variation, utilizing directional cues to effectively distinguish between noise and textures. The HEAL network is trained, validated and tested under different ultra-sparse configurations of 60 views and 30 views, demonstrating its advantages in reconstruction accuracy and detail enhancement.

## 1. Introduction

Computed tomography (CT) scanning technology enables non-invasive detection of internal structures and is widely used in diagnostic imaging and clinical radiotherapy [1,2]. Prolonged exposure to X-rays can cause radiation damage to the human body and increase the risk of diseases [3,4]. Therefore, the demand for reducing X-ray radiation dose during CT imaging is becoming increasingly urgent. Among methods for dose reduction, sparse-view CT imaging is an efficient approach. Over the past decade, many iterative algorithms based on compressive sensing (CS) [5,6] have been applied to sparse-view CT imaging. The most representative methods are based on total variation (TV) models, such as TV-minimization projection onto convex sets (TVM-POCS) [7] and adaptive steepest descent projection onto convex sets (ASD-POCS) [8]. These methods effectively suppress sparse-view artifacts, but TV constraints may cause loss of some detailed features and generate blocky appearance in noisy cases. Dictionary learning-based compressive sensing models are a superior sparsification method which can extract image-related bases for sparse representation of images, further improving the image quality of sparse imaging [9]. Although compressive sensing-based methods can reduce the number of samples in CT reconstruction, they are still far from meeting the desire for dose reduction in practical applications.

In recent years, deep learning has been widely applied in the field of CT imaging and has achieved some encouraging results [10,11,12,13], especially in sparse-view reconstruction, showing better imaging results than compressive sensing models [14,15]. Currently, deep learning-based sparse-view reconstruction methods can be categorized into four types: single-domain learning, direct mapping between measurement data and reconstructed images using networks, network models based on iterative reconstruction algorithms and dual-domain learning. Single-domain learning refers to image enhancement in the projection domain or image domain. Projection domain methods are mainly divided into two steps: pre-processing of sinogram using CNN networks [16,17], and then obtaining the reconstructed CT images through FBP and other reconstruction algorithms. These methods directly interpolate the sinogram using the powerful fitting ability of deep networks to obtain more complete projection data. However, the small errors introduced by direct manipulation of the sinogram may result in severe artifacts in the reconstructed image. Image domain methods use various neural networks for post-processing of reconstructed images. These neural network models can be supervised models, such as FBPConvNet based on U-net [10], RED-CNN based on encoder–decoder CNN [11], DD-net using dense blocks instead of convolutional layers [14], Framing U-Net overcoming the frame condition [18], and U-net with multi-level wavelets [19]. In addition to supervised models, there are also unsupervised or self-supervised models, such as the score-based unsupervised generative model proposed by Guan et al. [20] and the SCOPE self-supervised network [21] based on a novel projection strategy. Image domain methods can process CT reconstruction images quickly and are easy to deploy, but they may lead to unstable and inaccurate results due to the neglect of consistency with the original measurement data, which is unfavorable for clinical diagnosis [22]. The second type is direct mapping between measurement data and reconstructed images using networks [23,24]. For example, iRadonMAP [24] adopts fully connected layers to transform projection data into CT images based on the FBP algorithm. These models have good noise resistance, but the deep network is prone to various instabilities, making it difficult to directly apply them in practice. The third type is inspired by traditional iterative algorithms and unfolds the iterative reconstruction algorithm into a network, constructing regularization terms based on network models. Representative methods include the LEARN network based on expert evaluation [22], the Alternating Direction Method of Multipliers (ADMM) network [25], the RegFormer network based on local–nonlocal regularization [26] and the Deep Embedding-Attention-Refinement model which incorporates projection data and image prior knowledge into an analytic iteration model [27]. These methods can avoid the adjustment of regularization weight parameters and achieve significant performance improvements. However, due to the need to construct iterative processes similar to iterative reconstruction, they incur significant computational and memory resource costs. Additionally, the number of iterations directly affects the final performance in practical applications. The fourth type is dual-domain learning, which utilizes information from both the projection domain and the image domain to jointly reconstructs the image. In some network models, training is first conducted in the projection domain, and then the network is trained in the image domain after reconstruction. For instance, Hu et al. independently train cone-beam data in dual domains, achieving better results than single-domain training [28]. Lee et al. replace convolutional layers with wavelet transform and performed optimized reconstruction in the projection domain, image domain and dual domains, respectively [29]. The experimental results indicated that dual domain has the best artifact-suppression effect. DRONE employs a multi-stage optimization strategy, combined with residual learning and compressed sensing post-processing, which ensures certain accuracy while suppressing sparse artifacts [30]. However, these models trained separately in dual domains do not consider the potential interaction between the domains, and fail to fully exploit the benefits of hybrid domains. On the other hand, some other networks that combine domains with inter-domain consistency have shown impressive performance. For example, Sam’net introduces multi-level self-constraints and self-enhancement based on the dual domain [31], CLEAR integrates dual-domain learning into the WGAN-GP modality [32], WNet incorporates a learnable reconstruction layer [33]. These excellent works demonstrate the improvement brought by the mutual constraints of dual-domain joint learning. Additionally, researchers have made numerous attempts to achieve better results. For instance, Wu et al. add a skip-encode structure to the U-Net network to address its inherent shortcomings and reduce information loss [34]. This also demonstrated that improvements to U-Net can enhance the task of sparse reconstruction. These excellent deep learning methods partially address the problem of sparse view sampling, but when faced with the challenge of ultra-sparse view imaging (less than 100 views), there is still room for improvement in restoring the details and fine textures in the reconstructed images.

Inspired by the dual-domain joint learning, we propose a high-frequency enhanced and attention-guided learning network (HEAL). HEAL adopts a progressive improvement strategy to divide the image quality enhancement into two parts. The first part adopts a dual-domain joint optimization model as the backbone, and introduces attention mechanisms into the feature-scaling process to compensate for information loss incurred during the scale transformation operations conducted to acquire multiscale features. Furthermore, a constraint on the high-frequency component based on directional total variation is introduced to effectively differentiate between noise and texture features, simultaneously enhancing the network’s capacity for detail recovery and noise suppression. The second part involves an adversarial generative network in the image domain, focusing on transferring the statistical characteristics of real images, particularly high-frequency detail features, to the images generated by the network in the first part. This subnetwork aims to further enhance HEAL’s ability to recover high-frequency detail features.

The main contributions of our work are summarized as follows:

(1) We introduce a dual-domain progressive enhancement module in sparse-view CT, which utilizes the fidelity constraints of each domain as well as the consistency constraints between the domains to narrow the solution space. We also use a progressive enhancement strategy to decompose the sparse-view enhancement task, reducing the optimization pressure of each domain.

(2) We introduce an attention-guided mechanism into the feature-scaling process of the network, which includes channel attention and spatial attention. The introduction of this mechanism helps to compensate for the information loss incurred during the scale transformation operations conducted to obtain multiscale features.

(3) We propose a high-frequency component enhancement regularization term based on the combination of residual learning and direction-weighted total variation, effectively distinguishing noise and image textures and effectively enhancing the extraction and restoration of high-frequency information in images while suppressing noise.

The rest of this paper is organized as follows. We first introduce the overall framework of HEAL and the design details of feature scaling with attention mechanism and the high-frequency enhancement constraints based on direction-weighted total variation in Section 2. In Section 3, we present the quantitative and qualitative analysis of the experimental results. In Section 4, we discuss the advantages and limitations of our approach and some related work, and draw conclusions.

## 2. Methods

The main goal of this study is to develop a high-quality reconstruction model for ultra-sparse-view CT, focusing on two objectives, image fidelity and detail enhancement. To achieve these two objectives, we first establish a dual-domain progressive enhancement module (DDPM), which incorporates feature scaling with an efficient attention mechanism (FSAM) and a high-frequency component enhancement regularization term (HFER). Then, we introduce a detail enhancement moddule in the image domain. In the following sections, we first describe the overall framework and hierarchical structure of HEAL in Section 2.1, and then we describe the FSAM and HFER in detail in Section 2.2 and Section 2.3, respectively.

### 2.1. Overall Structure of HEAL Network

#### 2.1.1. Dual-Domain Optimization Module

**Figure 1:**
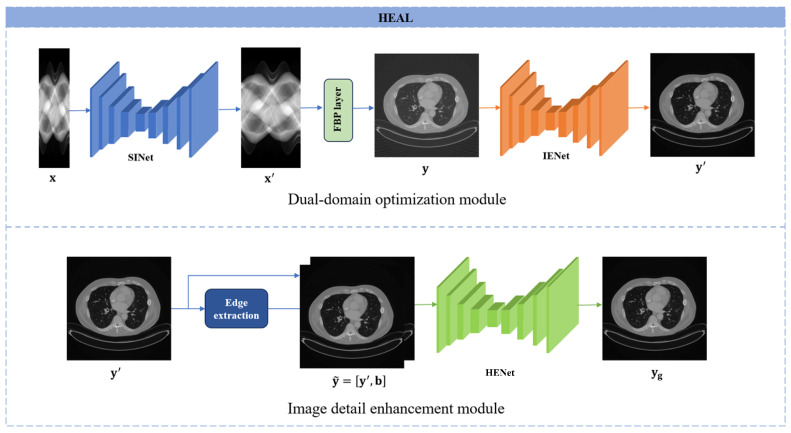
The overall architecture of HEAL. The dual-domain optimization module generates prior image $y^{\prime }$ for further optimization by the detail enhancement module.

**Figure 2:**
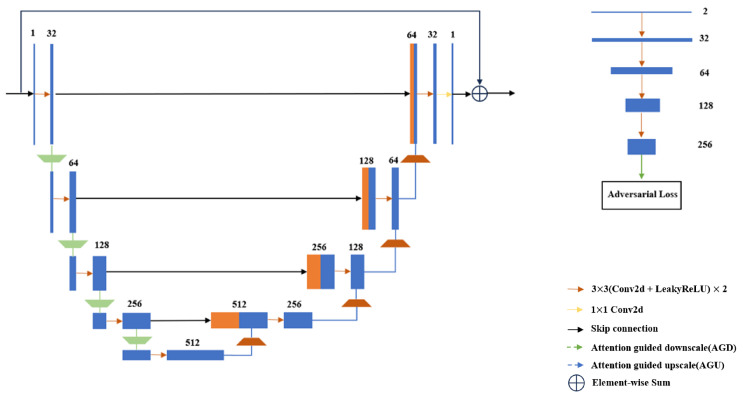
The left figure shows the network structure guided by the attention mechanism for feature scaling, and the right figure shows the discriminator network structure.

Figure 1 presents the flowchart of the HEAL network, starting with the dual-domain progressive enhancement module. For underdetermined problems like sparse-view CT imaging, traditional compressed sensing models have proven that enhancing the reconstruction image quality can be achieved by adding prior regularization terms to narrow the solution space. Moreover, introducing more prior terms is more likely to improve the reconstruction image quality. Therefore, in this study, a reconstruction layer is introduced to enable dual-domain learning. The advantage of dual-domain learning lies in its ability to simultaneously utilize fidelity constraints from both domains and the consistency constraint between the two domains to narrow the solution space. DDPM consists of the projection domain interpolation network (SINet) and the image domain enhancement network (IENet) in a cascaded manner. SINet is used to expand the initial sparse projection data to 180 views, and the IENet is used to further enhance the reconstructed image obtained from the 180 projection views to the reconstructed image obtained from the standard number of projection views, which is set to 720 in this paper. By adopting this progressive enhancement approach and distributing the complex ultra-sparse view reconstruction task between two networks, the burden on a single network is reduced. Both SINet and IENet adopt a modified U-Net network, as shown in Figure 2. U-Net can effectively extract detailed features at different scales, enhancing the detail-recovery capability of both networks. To further enhance the network’s detail-recovery capability, we introduce attention-guided mechanism to the upscale and downscale processes of the traditional U-Net. The detailed contents will be described in Section 2.2. The dual-domain network model can be represented as follows:

(1) $$\begin{array}{c} y^{\prime }=\Phi_{IE}\left(P\left(\Phi_{SI}\left(x\right)\right)\right), \end{array}$$

where *P* represents the FBP process with 180 views.

In this module, we establish multiple objective functions. Firstly, we have the L1 loss and between the generated images $x^{\prime }$, $y^{\prime }$ and their corresponding labels $x_{L}$, $y_{L}$ in the projection domain and image domain, respectively. Secondly, we introduce the dual-domain consistency loss:

(2) $$\begin{array}{c} L_{\mathrm{Consistency}\;}=\frac{1}{N}\sum_{i=1}^{N}\left|P^{\prime }{\left(y^{\prime }\right)}^{i}-{x_{L}}^{i}\right|, \end{array}$$

where $P^{\prime }$ denotes the forward projection process with 180 views, and *i* means the pixel index. This consistency loss can be utilized to highlight which features in the optimization process of the projection domain have a greater impact on the reconstruction results. Therefore, it can be considered as a strengthening constraint for the optimization of the projection domain, guiding the generation process in the projection domain. The overall objective function in this module can be formulated as follows:

(3) $$\begin{array}{c} Loss=\lambda_{1}L_{1}\left(x^{\prime },x_{L}\right)+\lambda_{2}L_{1}\left(y^{\prime },y_{L}\right)+\lambda_{3}L_{Consistency}+R_{HF}\left(y^{\prime },y_{L}\right), \end{array}$$

where $\lambda_{1}$, $\lambda_{2}$ and $\lambda_{3}$ are weight coefficients, and $R_{HF}$ is a novel residual-based detail enhancement constraint. A detailed description of $R_{HF}$ is in Section 2.3.

#### 2.1.2. Image Detail Enhancement Module

In the task of ultra-sparse reconstruction, obtaining preliminary interpolated projection images that meet the requirements for image reconstruction is crucial. In the aforementioned dual-domain module, the image domain network can be regarded as one of the regularization terms of the projection domain network. By amplifying the fitting error in the projection domain through the reconstruction image with enriched details and significant feature expression, the projection domain can be regularized accordingly. Through dual-domain training, the fitting performance in the projection domain can be greatly improved. To further enhance the detail-recovery capability in the image domain, we incorporated an image detail enhancement module based on generative adversarial networks after the dual-domain network. The objective of this network is to transfer the high-frequency statistical characteristics from real reconstructed images to the generated images. The objective of this network is to transfer the high-frequency statistical characteristics from real reconstructed images to the generated images. The structure of the generator HENet is consistent with the previous dual-domain module, while the discriminator is shown in Figure 2. To enhance the network’s emphasis on detail components, edge extraction is performed on the input images before they are fed into the generator. These edges, along with the original images, are utilized as multimodal data during network training. The edge extraction process can be described as follows:

(4) $$\begin{array}{c} b=\left|F\left(y^{\prime },s_{H}\right)\right|+\left|F\left(y^{\prime },s_{W}\right)\right|, \end{array}$$

where *F* represents the edge extraction process, $s_{H}$ and $s_{W}$ are the Sobel operators for horizontal and vertical edge extraction, respectively. To avoid edge smoothing and detail loss caused by pixel-based mean squared error (MSE), and to achieve stable training, we choose a discriminator with a gradient penalty term and use Wasserstein distance as the measure of distribution difference [35,36,37]. The consistency loss and high-frequency enhancement constraint are also used as regularization terms for the generator to limit erroneous generation. In the training of the generative adversarial network, we optimize the generator and discriminator by maximizing and minimizing the following objective function, respectively:

(5) $$\begin{array}{c} \min_{G}\max_{D}L_{GAN}\left(G,D\right)=L_{MSE}\left(y_{L},G\left(C\left(y^{\prime },b\right)\right)\right)+R_{HF}\left(y_{L},G\left(C\left(y^{\prime },b\right)\right)\right)+L_{Consistency}+\\ \lambda_{WGAN}\left(E_{\left(y^{\prime }\right)}\left[D\left(G\left(C\left(y^{\prime },b\right)\right)\right)\right]-E_{y_{L}}\left[D\left(y_{L}\right)\right]+\mu E_{\hat{y}}\left[{\left(\nabla_{\hat{y}}D\left(\hat{y}\right)-1\right)}^{2}\right]\right), \end{array}$$

where $\lambda_{WGAN}$ are weight coefficients, *C* is the cascade operator, $\hat{y}$ is obtained by uniformly sampling from the predicted image and the corresponding real sample, and μ is the coefficient of the gradient penalty term, set to 10 in this study. The refined result $y_{g}$ from the generator network can be represented as follows:

(6) $$\begin{array}{c} y_{g}=\Phi_{HENet}\left(y^{\prime },b\right). \end{array}$$

### 2.2. Feature Scaling with Attention Mechanism

The dual-domain network in this paper is modified based on U-Net. The traditional U-Net changes the receptive field by upscale and downscale, thereby extracting features at different scales. However, it inevitably loses information during the downscale and upscale processes, leading to a decrease in network performance [38]. Based on this observation, it can be inferred that optimizing the feature-scaling process of U-Net to improve its performance is feasible.

Recent research on high-level visual tasks shows that there is a certain hierarchy in both feature channels and visual primitives, and the information loss during the feature-scaling process can be compensated by introducing attention mechanisms [39,40]. Relevant practices have already been applied in low-level visual tasks like denoising [40]. Therefore, to enhance the detail-recovery capability of projection domain and image domain networks, we introduce feature-scaling modules with attention mechanisms into the upscale and downscale process in U-net, namely the AGU and AGD modules shown in Figure 2. The attention-guided feature scaling consists of two parts: channel attention-guided scaling and spatial attention-guided scaling.

**Figure 3:**
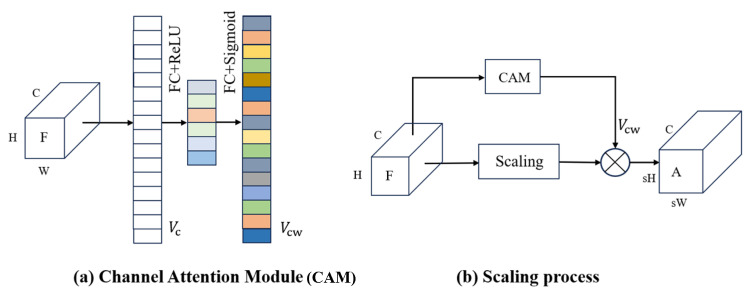
Channel Attention Module (CAM) and feature-scaling process guided by attention. In (**a**), the input is a feature map with C, H, W representing the number of channels, height and width of the feature map, respectively. After global average pooling, C internal global feature values are obtained. (**b**) represents the feature-scaling process guided by channel attention, where weighted processing is performed channel-wise.

As the structure shown in Figure 3a, firstly, we use global average pooling to obtain channel feature vector $V_{c}$ from the feature map *F*. Then, $V_{c}$ is encoded through fully connected layers and ReLU transformation. Followed by decoding through fully connected layers and Sigmoid transformation, the weight vectors $V_{c}w$ describing the importance of each channel is generated. The scaled result *A* guided by channel attention is shown in Figure 3b.

**Figure 4:**
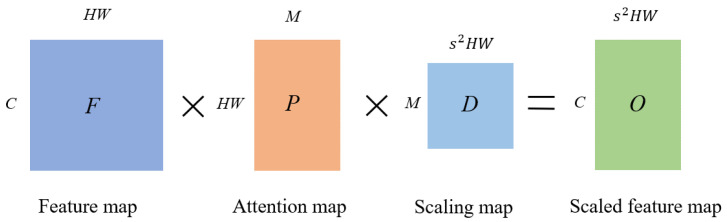
Spatial Attention Module (SAM) and the feature-scaling process guided by attention. From left to right, the input feature map *F*, the attention map *P* that gathers all the feature map pixels, the scaling map *D* that adaptively allocates spatial bases and the scaled feature map *O*.

Channel attention only focuses on the differences in importance between channels, ignoring the differences in importance between different positions within a channel. Therefore, we introduce the spatial attention-guided scaling, as shown in Figure 4. For ease of description, we reshape the feature map to 2-dimension space, denoted as $F={\left[f_{1},\ldots ,f_{C}\right]}^{T},f_{i}\in R^{HW\times 1}$. Since the main visual primitives in the image tend to keep similar [39], we introduce the attention map $P=\left[p_{1},\ldots ,p_{M}\right],p_{i}\in R^{HW\times 1}$ to extract a set of spatial bases containing M visual primitives, denoted as $\mathrm{FP}=\left[{\mathrm{Fp}}_{1},\ldots ,{\mathrm{Fp}}_{M}\right]$, where ${\mathrm{fp}}_{i}\in R^{C\times 1}$ is a visual primitive. Then, by introducing the scaling map $D=\left[d_{1},\ldots ,d_{s^{2}HW}\right],d_{i}\in R^{M\times 1}$, the spatial bases are adaptively allocated to each position in the scaled features, resulting in the output under spatial attention guidance:

(7) $$\begin{array}{c} O=FPD. \end{array}$$

**Figure 5:**
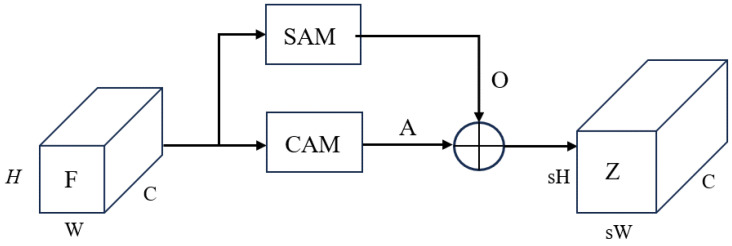
Flowchart of feature-scaling mechanism under attention guidance.

The feature-scaling process under attention guidance is shown in Figure 5, where the resulting $Z\in R^{C\times sH\times sW}$ can be expressed as:

(8) $$\begin{array}{c} Z=O+A. \end{array}$$

### 2.3. High-Frequency Enhancement Constraints Based on Direction-Weighted Total Variation

Because images contain more low-frequency components and fewer high-frequency components, neural networks are more likely to learn low-frequency features during training, resulting in better performance in recovering low-frequency components [41,42]. In ultra-sparse reconstruction, the key challenge is recovering high-frequency details. We address this by introducing a high-frequency enhancement constraint based on direction-weighted total variation of the residual image. The residual image is represented as $z=(y^{\prime }-y_{L})$, where $y^{\prime }$ is the predicted image and $y_{L}$ is the label image. In residual images, due to the higher proportion of high-frequency components, introducing constraints on residual images allows the network to learn high-frequency features supplementarily, thereby improving performance in recovering high-frequency components. The straightforward approach is to apply a total variation model to smooth the residual image. However, the residual image contains both valuable detail and texture as well as irrelevant high-frequency noise. To address this issue, we construct the following direction-based total variation model to enhance the network’s ability to extract fine details and textures from the residual image. First, we need to obtain the first-order gradients in the horizontal and vertical directions for each point on the residual image as follows:

(9) $$\begin{array}{c} g_{h}=z\left(n_{w},n_{h}\right)-z\left(n_{w},n_{h}-1\right), \end{array}$$

(10) $$\begin{array}{c} g_{w}=z\left(n_{w},n_{h}\right)-z\left(n_{w}-1,n_{h}\right). \end{array}$$

Then, the gradient magnitude and direction at each point are obtained:

(11) $$\begin{array}{c} nor_{hw}=\sqrt{g_{h}^{2}+g_{w}^{2}}, \end{array}$$

(12) $$\begin{array}{c} \theta_{hw}=tan^{-1}\frac{g_{h}}{g_{w}}. \end{array}$$

Due to the higher consistency of noise compared to texture features in different directions, the gradient differences in various directions can be utilized to distinguish textures from noise. Therefore, we introduce the magnitude of the gradient’s vertical direction:

(13) $$\begin{array}{c} ver=\left|resample(\theta_{hw})\right|, \end{array}$$

where resample signifies the resampling of the gradient’s vertical direction. By comparing the magnitudes of the gradient direction and its vertical direction vector, a descriptor that characterizes the difference in directional gradient consistency of the pixel can be obtained:

(14) $$\begin{array}{c} \alpha =\frac{nor}{ver}. \end{array}$$

Here, the α value measures noise and texture prominence. An α value close to 1 indicates good directional gradient consistency, which is likely noise. An α value far from 1 suggests poor consistency, which is likely textures. Based on understanding we can develop the frequency enhancement constraint as follows:

(15) $$\begin{array}{c} R_{HF}=\frac{1}{N_{w}N_{h}}\sum_{i=1}^{N_{w}N_{h}}\alpha_{i}nor_{i}. \end{array}$$

## 3. Experiments and Results

### 3.1. Implementation and Training Details

The network was implemented using the Pytorch framework. All experiments were conducted on a computer equipped with an NVIDIA GeForce RTX 3090 graphics card and an Intel(R) Core(TM) i5-11400 processor. The weights $\lambda_{1}$, $\lambda_{2}$, $\lambda_{3}$ and $\lambda_{WGAN}$ for the loss functions were all set to 1, and μ was set to 10. The Adam algorithm [43] was used for optimization. In the dual-domain progressive enhancement module, $\beta_{1}$ and $\beta_{2}$ were set to 0.9 and 0.999, respectively. In the Image detail enhancement module, $\beta_{1}$ and $\beta_{2}$ were set to 0.5 and 0.9, respectively. The number of visual primitives *M* was set to 96. The learning rate started from 1 × $10^{-3}$ and decayed to 1 × $10^{-5}$ The batch size was set to 2, and the number of epochs was set to 30. To demonstrate the performance of the proposed method, FBPConvNet [10], DDNet [14], RegFormer [26] and DRONE [30] were used as comparisons. Three commonly used objective image quality assessment metrics are used for quantitative evaluation of reconstruction performance: root mean square error (RMSE), peak signal-to-noise ratio (PSNR) and structural similarity index (SSIM) [44]. Additionally, we have introduced two assessment metrics in our experiments that have been demonstrated to be consistent with subjective evaluations [45], namely Visual Information Fidelity (VIF) [46] and Gradient Magnitude Similarity Deviation (GMSD) [47]. Higher values of PSNR, SSIM and VIF, and lower values of RMSE and GMSD indicate better performance.

### 3.2. Experimental Data Preparation

The dataset used in the experiment is from https://public.cancerimagingarchive.net/nbia-search/ (accessed on 10 October 2022). The dataset contains CT data from a total of 32 patients. We selected data from 28 patients for network training and reserved data from 4 patients for testing. the projection process was conducted using fan-beam geometry, with the projection angle ranging from [0, 2π]. The distances from the X-ray source to the detector and the rotation center were set to 64.5 cm and 32.25 cm, respectively. The detector array consisted of 736 detector elements, with each element having a length of 0.3 mm. The number of standard views was set to 720, and the numbers for sparse views were set to 30 and 60.

### 3.3. Results

**Figure 6:**
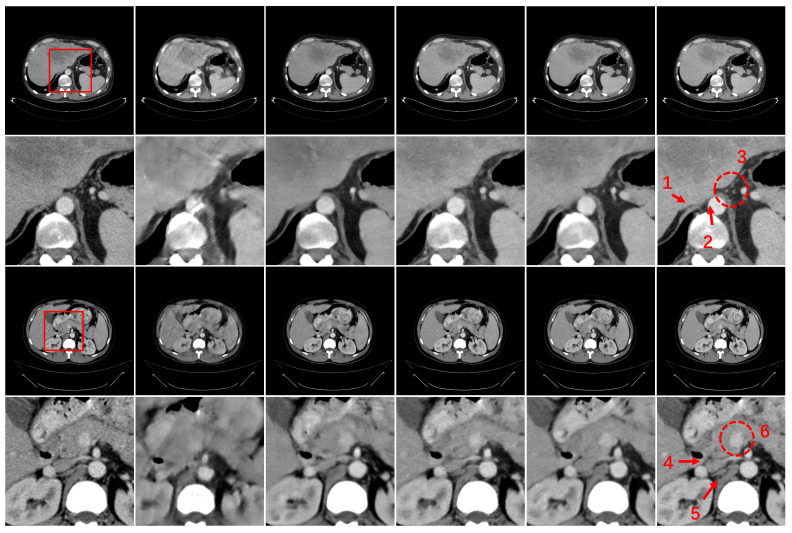
Reconstruction results for Case 1 and Case 2. Column 1 is the reconstruction results of FBP based on 720 views, which can be considered as the reference results. Columns 2 to 6 from left to right represent the results of DDNet, FBPConvNet, DRONE, RegFormer and HEAL based on 60 views. The second and fourth rows show the ROIs corresponding to the areas surrounded by the red boxes. The display window of the images is [−140 290] HU.

Figure 6 show representative Reconstruction results using different methods for 60 views. As shown in Figure 6, DDNet exhibits noticeable artifacts and significant loss of image details. Compared to DDNet, FBPConvNet provides better artifact removal capability, but the resulting images appear overly smooth with insufficient detail restoration. In contrast to the single-domain learning networks of DDNet and FBPConvNet, DRONE and RegFormer combine dual-domain constraints to improve image quality, resulting in significant improvements in image details and edge clarity. However, when compared to the reference image, the resulting images from DRONE and RegFormer still exhibit some blurring. By comparing with the competing methods, the proposed HEAL framework demonstrates the best performance in image quality. To further demonstrate the advantages of HEAL, regions of interest (ROI) are selected and magnified for a more detailed comparison. In Case 1, as indicated by arrows “1” and “2”, FBPConvNet, DRONE and RegFormer all achieve better structural restoration compared to DDNet. However, there are still some blurring of boundaries. In comparison, HEAL provides clearer organ boundaries and tissue details. In the region marked by circle “3”, DDNet and FBPConvNet fail to restore these fine structures, while DRONE and RegFormer partially addressed this issue. However, these fine structures still exhibit noticeable blurring. HEAL demonstrates strong capability in detail restoration. This capability is more pronounced as the complexity of image features increases. For example, in Case 2, the regions indicated by arrows “4” and “5” exhibit structural deficiencies in the reconstruction images of DDNet and FBPConvNet, making them unable to provide clinically useful image features. RegFormer shows more noticeable details but also exhibits structural deficiencies. In comparison, DRONE shows more pronounced detail features than the previous three methods, but in cases where multiple small structures are densely arranged in a small region, there are distortions and blurring in the boundaries of these small tissue structures. In terms of structural fidelity, as shown in circle “6”, DRONE and RegFormer restore the missing features in DDNet and FBPConvNet, but there are still distortions in the structure. The proposed HEAL maintains considerable consistency in structure and obtains relatively clear edges.

**Figure 7:**
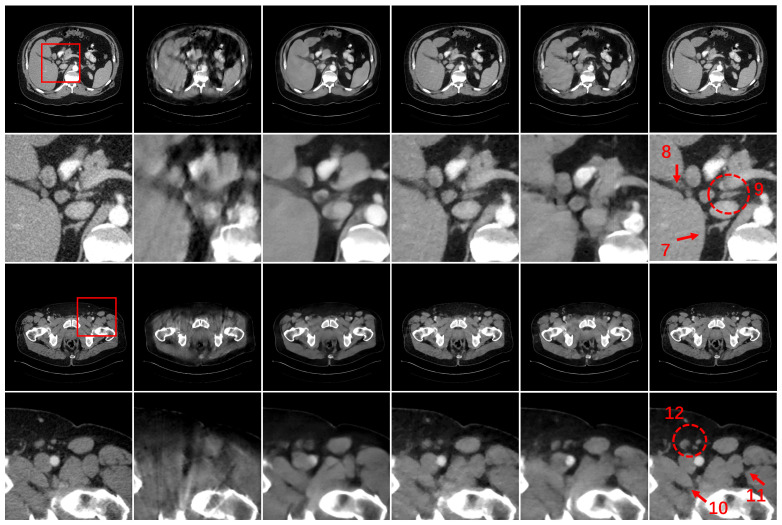
Restoration results for Case 3 and Case 4. Column 1 is the reconstruction results of FBP based on 720 views, which can be considered as the reference results. Columns 2 to 6 from left to right represent the results of DDNet, FBPConvNet, DRONE, RegFormer and HEAL based on 30 views. The second and fourth rows show the ROIs corresponding to the areas surrounded by the red boxes. The display window of the images is [−140 290] HU.

To validate the restoration performance of HEAL under ultra-sparse conditions, we also conducted comparative experiments with 30 views. From the results shown in Figure 7, we can see that the resulting images of DDNet exhibit more sparse-view artifacts, the resulting images of RegFormer also show noticeable artifacts and the resulting images of FBPConvNet exhibit more structural distortions. In contrast, DRONE demonstrate the best performance in artifact removal and detail fidelity among all comparative methods, thanks to its dual-domain constraints. By comparing the marked regions in the enlarged images, it is intuitive that the resulting images restored by HEAL under the condition of 30 views outperform DRONE in terms of both detail and structural fidelity.

To quantitatively compare the performance of the proposed method and the competing methods, we conducted quantitative analyses of the resulting images under both 60 and 30 views. The quantitative analysis results are shown in Table 1. It is evident from the table that DRONE and RegFormer outperform DDNet and FBPConvNet. Additionally, the proposed HEAL method achieves the smallest RMSE and GMSD values and the largest SSIM, PSNR and VIF values, indicating superior image restoration compared to the comparative methods. This conclusion aligns with the qualitative analysis conducted earlier.

For a more comprehensive comparison and evaluation of all sparse reconstruction methods used in previous experiments, we analyzed the training time required for each method to converge and the inference time needed for a single image under the condition of 60 projection views. The test results are presented in Table 2. Regarding the training time, DDNet and FBPConvNet, based on single-domain learning, require the shortest training time. DRONE needs a longer training time due to the necessity of training four separate networks. RegFormer takes the longest time because the iterative process is added to the training process. The HEAL model proposed in this paper employs dual-domain joint learning and an adversarial generative network, leading to a relatively longer training time. In terms of inference time for a single image, DDNet and FBPConvNet require the shortest time, but their image reconstruction effect is poor. The DRONE method, due to its use of iterative post-processing, has the longest inference time. The RegFormer method performs better in image reconstruction and inference speed at 60 views compared to DRONE, but as mentioned earlier, the reconstruction effect of RegFormer is slightly worse at 30 views. In a comprehensive comparison, the HEAL model proposed in this paper achieves the best balance between image reconstruction effect and inference speed.

### 3.4. Ablation Study

In this part, we conducted an ablation experiment to validate the effectiveness of dual-domain progressive enhancement module, feature scaling with efficient attention mechanism, high-frequency component enhancement regularization term and image detail enhancement module in the HEAL network.

**Figure 8:**
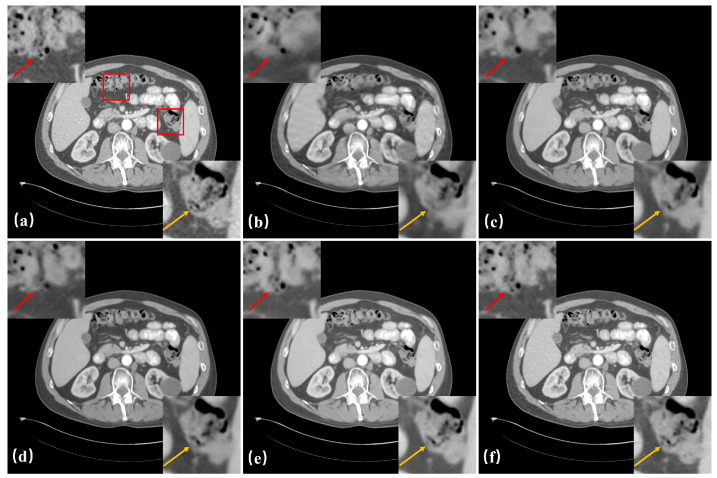
Reconstruction results of different modules with 60 views and the ROIs corresponding to the areas surrounded by the red boxes. (**a**) Ground truth, (**b**) Results of dual-domain training separately, (**c**) Results of dual-domain joint training using traditional U-net, (**d**) Results with the addition of feature-scaling module guided by attention based on (**c**), (**e**) Results with the addition of residual high-frequency constraint term based on (**d**), (**f**) Results of edge-enhanced adversarial network. The display window of the images is [−180 280].

Figure 8 shows the reconstruction results after adding each module under 60 views. From Figure 8c, it can be seen that by fully utilizing the fidelity constraints within each domain and the consistency constraints across domains, the dual-domain joint learning model effectively narrows the solution space. Consequently, in terms of artifact removal and image structure restoration, the reconstructed images are superior to those of the separately trained dual-domain model (Figure 8b). However, the reconstructed images in Figure 8c still exhibit excessive smoothness and detail distortion compared to the ground truth. This issue improves with the successive addition of the attention-guided feature-scaling module and high-frequency enhancement regularization term. Specifically, with the addition of attention guidance to the feature-scaling module, the lost information during the scale transformation is recovered, making the structures in the reconstructed images more consistent with the ground truth, as shown in the enlarged region in Figure 8d. The high-frequency enhancement constraint based on direction-weighted total variation distinguishes between image textures and noise, further enhancing the network’s ability to capture details and significantly improving the model’s performance in restoring small details, as shown in the enlarged region in Figure 8e. With these improvements, the dual-domain joint learning model provides better image detail restoration capabilities. To further improve image quality, the image detail enhancement module leverages the powerful generative ability of generative adversarial networks, combined with image edge information, resulting in clearer tissue edges and richer image details, as shown in Figure 8f. As seen in Table 3, the results of the above qualitative analysis are consistent.

## 4. Discussion and Conclusions

Sparse-view CT imaging is an effective solution that can significantly reduce radiation dosage. Compressed sensing theory has demonstrated the feasibility of obtaining ideal reconstructed images through sparse sampling. However, practical implementation has shown that significant reduction in sampling views cannot be achieved. The application of deep learning models has greatly reduced the sampling views while significantly improving the image quality of sparse reconstruction. However, current deep learning-based sparse reconstruction models are mainly extensions of conventional models used in natural image processing, without special strategy designs focusing on the key aspects of sparse reconstruction. Therefore, there is still considerable room for improvement in detail recovery in reconstructed images. The HEAL model introduced in this paper, employs a suite of detail enhancement strategies to bolster image detail recovery in sparse reconstruction. Comparative studies conducted under 60 and 30 views demonstrate the model’s superior performance over competing methods, particularly in the restoration of image details. The acquisition of these effects is primarily attributed to the application of several strategies. Firstly, the dual-domain joint optimization model enhances the effect of dual-domain models on detail recovery through both intra-domain fidelity constraints and inter-domain consistency constraints, which was initially validated in the ablation study. Secondly, the introduction of attention mechanisms during the feature-scaling process can compensate for the important information loss caused by scale transformation operations. Thirdly, the combined use of residual images and directional total variation effectively prevents noise interference in the process of extracting texture features by the network. It can be clearly seen in the ablation experiments that the addition of this strategy significantly improves the network’s detail-recovery capability. Lastly, the edge-aware WGAN network model further enhances the network’s detail-recovery capability due to the powerful generative ability of adversarial generative networks and the assistance of high-frequency information related with image boundaries.

Our study indicates that by introducing specific strategies tailored for sparse reconstruction, it is indeed possible to effectively enhance the performance of sparse reconstruction networks, providing valuable insights for further optimization in this area. Since the model architecture utilized in this study is still relatively simple, based on the UNet architecture, we speculate that employing more complex architectures such as transformer or newly emerging diffusion architectures could further improve sparse reconstruction performance. However, this may come at the cost of increased computational and time costs. Therefore, the HEAL model presented here, based on a simple model architecture, still retains efficiency advantages in practical applications. One significant limitation of this model in practical applications is its reliance on the dual-domain joint optimization strategy, which requires the introduction of reconstruction layers between the projection domain and image domain, making it less adaptable to cone-beam sparse reconstruction. To extend its applicability to cone-beam sparse reconstruction, apart from significantly increasing memory usage, a more feasible strategy would be to utilize separately trained dual-domain strategies. However, this may result in a certain degree of performance degradation.

Finally, in this study, we also discovered an interesting phenomenon. Existing research has found that sparse reconstruction methods solely based on deep learning models produce images lacking in fidelity, leading them to incorporate iterative processes after the network [30]. However, in our study, we also attempted to integrate iterative processes into the subsequent stages of the network and found little improvement in both qualitative and quantitative evaluations (as the addition of iterative processes did not effectively enhance the reconstruction results, we did not present this result in the paper). This indicates that our model exhibits very high fidelity.

In summary, we propose an innovative high-frequency enhanced and attention-guided learning network (HEAL) to address the sparse-view reconstruction problem. HEAL adopts a series of specially designed optimization strategies to improve the performance of the sparse reconstruction model. Through experiments conducted under ultra-sparse configurations of 60 views and 30 views, HEAL demonstrates its advantages in terms of reconstruction accuracy and detail enhancement.

## Figures and Tables

**Table 1:** Quantitative evaluation of the reconstruction performance of different methods.

Views	Metrics	DDNet	FBPConvNet	DRONE	RegFormer	Ours
60 views	SSIM	0.9549	0.9736	0.9797	0.9814	**0.9853**
	PSNR	39.88	43.76	45.29	45.92	**47.21**
	VIF	0.5857	0.7011	0.7536	0.7674	**0.8012**
	RMSE	6.981	2.856	1.990	1.718	**1.231**
	GMSD	0.0385	0.0185	0.0095	0.0086	**0.0071**
30 views	SSIM	0.9248	0.9587	0.9734	0.9663	**0.9802**
	PSNR	36.23	41.13	43.83	42.09	**45.60**
	VIF	0.4976	0.6255	0.6576	0.6354	**0.7043**
	RMSE	16.36	5.248	3.030	4.216	**1.854**
	GMSD	0.0595	0.0312	0.0198	0.0266	**0.0122**

**Table 2:** Training time to convergence and inference time for a single image of different methods.

Phase	DDNet	FBPConvNet	DRONE	RegFormer	Ours
Training (h)	10.5	22.4	34	102	61
Inference (s)	0.01	0.03	96	0.28	0.08

**Table 3:** Quantitative analysis of different modules under 60 views.

DDPM	FSAM	HFER	HENet	SSIM	PSNR	RMSE
-	-	-	-	0.9744	44.14	2.643
✓	-	-	-	0.9791	44.63	2.345
✓	✓	-	-	0.9812	45.50	1.937
✓	✓	✓	-	0.9833	47.07	1.336
✓	✓	✓	✓	0.9853	47.21	1.231

## Data Availability

The dataset used in the experiment is from CancerImage.
